# Amazonian Triatomine Biodiversity and the Transmission of Chagas Disease in French Guiana: *In Medio Stat Sanitas*

**DOI:** 10.1371/journal.pntd.0004427

**Published:** 2016-02-11

**Authors:** Julie Péneau, Anne Nguyen, Alheli Flores-Ferrer, Denis Blanchet, Sébastien Gourbière

**Affiliations:** 1 UMR 228 ESPACE-DEV-IMAGES, ‘Institut de Modélisation et d'Analyses en Géo-Environnement et Santé’, Université de Perpignan Via Domitia, Perpignan, France; 2 Laboratoire de Parasitologie-Mycologie, Centre Hospitalier de Cayenne and Faculté de Médecine, Equipe « Ecosystèmes Amazoniens et Pathologie Tropicale » (EA3593), Université de Antilles et de la Guyane, Cayenne, French Guiana; Universidad de Buenos Aires, ARGENTINA

## Abstract

The effects of biodiversity on the transmission of infectious diseases now stand as a cornerstone of many public health policies. The upper Amazonia and Guyana shield are hot-spots of biodiversity that offer genuine opportunities to explore the relationship between the risk of transmission of Chagas disease and the diversity of its triatomine vectors. Over 730 triatomines were light-trapped in four geomorphological landscapes shaping French-Guiana, and we determined their taxonomic status and infection by *Trypanosoma cruzi*. We used a model selection approach to unravel the spatial and temporal variations in species abundance, diversity and infection. The vector community in French-Guiana is typically made of one key species (*Panstrongylus geniculatus*) that is more abundant than three secondary species combined (*Rhodnius pictipes*, *Panstrongylus lignarius and Eratyrus mucronatus*), and four other species that complete the assemblage. Although the overall abundance of adult triatomines does not vary across French-Guiana, their diversity increases along a coastal-inland gradient. These variations unravelled a non-monotonic relationship between vector biodiversity and the risk of transmission of Chagas disease, so that intermediate biodiversity levels are associated with the lowest risks. We also observed biannual variations in triatomine abundance, representing the first report of a biannual pattern in the risk of Chagas disease transmission. Those variations were highly and negatively correlated with the average monthly rainfall. We discuss the implications of these patterns for the transmission of *T*. *cruzi* by assemblages of triatomine species, and for the dual challenge of controlling Amazonian vector communities that are made of both highly diverse and mostly intrusive species.

## Author Summary

A major aspect of the evolution of Life on Earth is the contemporary loss of biodiversity. To understand the effects of biodiversity on biological phenomena is increasingly important. A key question is whether biodiversity facilitates or reduces infectious diseases transmission. Surprisingly, although many human pathogens are transmitted by arthropods, we still know little about the impact of their diversity on disease risk. We focused our investigation on French Guiana, a hot-spot of biodiversity, and looked at the effect of triatomine vector diversity on the risk of Chagas disease transmission, a major zoonosis in Latin American. We collected bugs in various locations and times, and quantified their infection by *Trypanosoma cruzi*. We showed that triatomine diversity varies between landscapes and that intermediate levels of diversity lead to minimal risks of transmission. Accordingly, losing vector biodiversity could have positive or negative effects on Chagas disease risk, depending on the pre-existing level of such diversity. A second key result emerging from this study is that highly diverse vector community can lead to biannual peaks of transmission of Chagas disease, which places populations in a "double jeopardy" each year. This calls for a better assessment and account of triatomine biodiversity in public health policy.

## Introduction

Tropical and sub-tropical countries from all over the globe are vulnerable to two of the major scientific and socio-economic issues faced by today’s human societies; the loss of biodiversity and the burden of infectious diseases. These issues are linked one with another since a critical consequence of biodiversity loss on human well-being is its potential impact on the emergence and transmission of infectious diseases [1–3]. Zoonotic diseases spilling over from wild species to humans typically involve multiple hosts and/or vectors species, which happen to be the case for many of the Neglected Tropical Diseases (NTD) such as Chagas disease, leishmaniasis, Human African Trypanosomiasis, Onchocerciasis or Schistosomiasis. Understanding how changes in the diversity of those communities affect the transmission of human pathogens is then highly topical in the context of global changes [4–6], and is critical to the evaluation of ecosystem services and Eco-Health approaches intended to reduce the burden of NTD and other infectious diseases [7–9].

The effects of host biodiversity on transmission have been investigated for various human pathogens and comprehensive reviews have shown that, depending on the pathogens and/or the ecological contexts and scales, an increase in host species richness can either amplify or ‘dilute’ the transmission of infectious diseases to humans (see [10] for a review). Among NTD, host biodiversity seems to increase the transmission of Onchocerciasis and Schistosomiasis [10] and to reduce the risk of transmission of leishmaniasis [11], Chagas disease ([12,13] but see [14]) or Buruli Ulcer [15]. Together with various host species, there are often several hematophagous vector species involved in the transmission cycle of any given human vector-borne pathogen. There are about 30–40 species of Anopheles mosquitoes [16], 30 species of tsetse flies belonging to the *Glossina* genus [17], more than 20 species of sandflies [18], and up to 70 species of triatomine bugs [19] that are capable of transmitting malaria, African sleeping sickness, leishmaniasis and Chagas disease, respectively. The effect of such insect vector biodiversity has received much less attention despite vector-borne parasites being severely detrimental to human health, and while arthropod diversity is already responding to climate changes [20,21].

We investigated the effect of the diversity of triatomine species that are vectors of *Trypanosoma cruzi*, on the risk of transmission of Chagas disease. This parasitic disease, also known as the American trypanosomiasis, is a key NTD afflicting people of Latin America, with an estimated 6–7 million infected persons [22] and another 25 million at risk of infection [23]. The etiologic agent, *Trypanosoma cruzi*, is able to infect about half of the 140 known species of triatomines (Hemiptera: Reduviidae), all of these hematophagous species being suspected to transmit the parasite to a large diversity of vertebrate hosts (e.g. [19,24]). Although the fecal or ‘stercorarian’ transmission of *T*. *cruzi* makes the probability of host infection very low for any potentially infectious contact [25], vector transmission remains the main road of human infection as compared to congenital or oral transmission.

The major part of the ecology and control of the disease is focused on ‘domestic’ vector species that have adapted to human habitat and are able to set up colonies inside human dwellings [26 for a review]. In this context, vector species diversity is typically low, and vector control initiatives aim at reducing or eliminating triatomine house infestation by indoor insecticide spraying, which has proved to be efficient at various scales [27,28]. While such small vector species communities are representative of highly endemic areas, in many other places across Latin America the transmission of the disease is associated to ‘intrusive’ vectors (e.g. [26]). Although those vectors do not colonize houses, they can still transmit the disease to 1–5% (e.g. [29–31]) and up to 16% [32] of the population during their transitory stay inside human dwellings. In this entomological setting, where vector dispersal strongly connects the human and wild habitats in typical source-sink dynamics [33,34], vector diversity can be much higher as it reflects the biodiversity of the wild assemblages.

We focused our study on French-Guiana, one of the 21 areas endemic for Chagas disease [22,35], where 14 triatomines species have been previously reported [36]. This high species diversity represents a large fraction of over 27 recognized species of Amazonian triatomines [37]. In this ‘hot-spot’ of triatomine biodiversity, key domestic species are lacking and the vector community is made of intrusive species that are responsible for the transmission of the disease, whose prevalence can reach up to 7% in some communities [30]. Those are remarkable circumstances to investigate the effect of vector biodiversity on *T*. *cruzi* transmission. We combined longitudinal data on the abundance and diversity of triatomine species, molecular characterization of vector infection by *T*. *cruzi*, and mathematical modelling in order to infer the spatial and temporal variations in the species structure and the infectiveness of the vector community. We then combined these data to estimate the force of infection associated with each vector species and quantify their relative contributions to the overall risk of transmission of Chagas disease.

## Methods

### Study area

**Fig 1:**
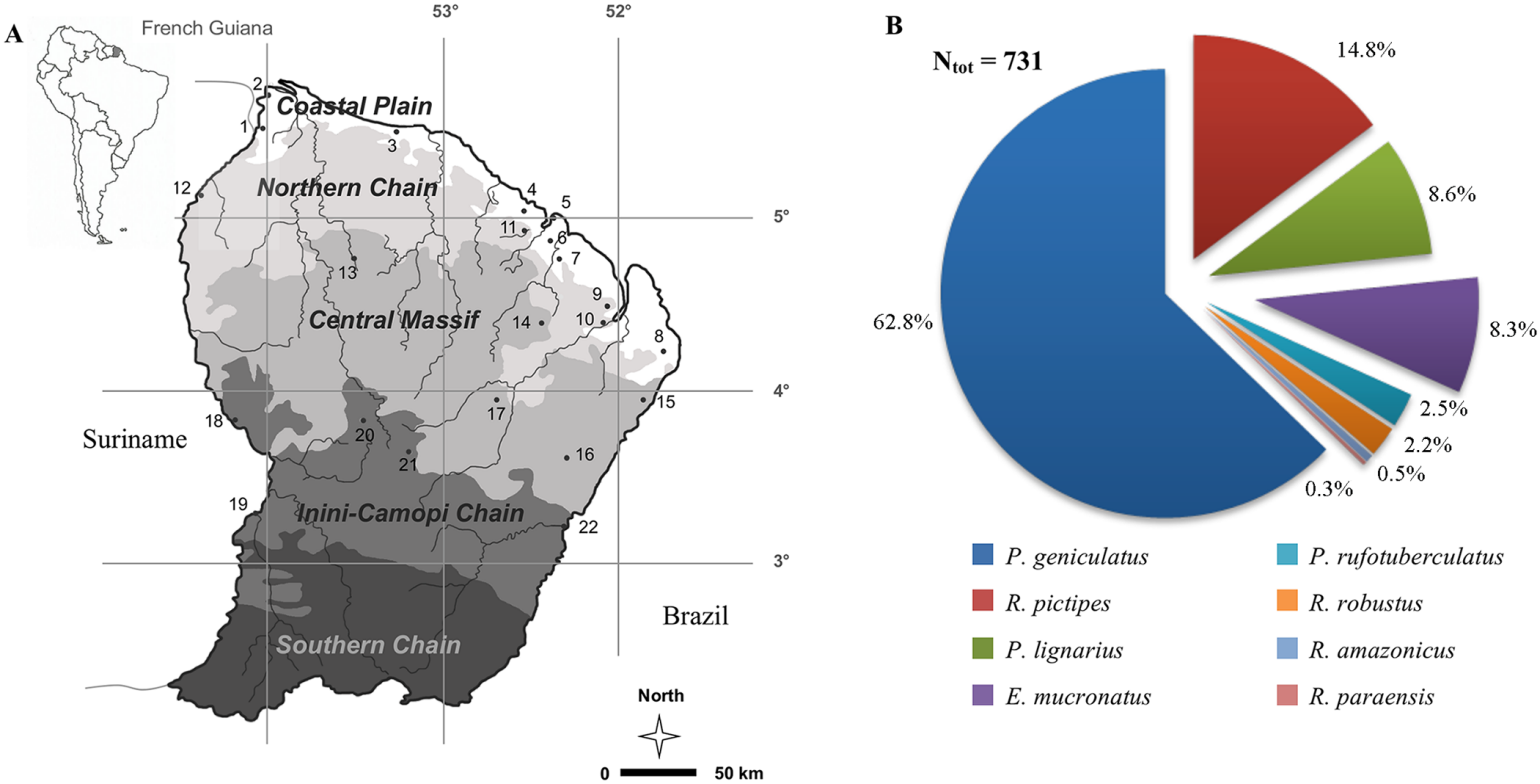
French Guiana and the biodiversity of its triatomine community. (A) French Guiana is partitioned into five geomorphologic landscapes: the Coastal Plain (CP), the Northern Chain (NC), the Central Massif (CM), the Inini-Camopi chain (IC), and the Southern chain. Black circles stand for the 22 field sites where triatomines were captured. A list of these sites with their GPS coordinate is provided in S1 Table. (B) The 731 adult triatomines captured across the four sampled landscapes belonged to 8 species. The colour legend used to refer to each of these species (in all figures of the paper) appears at the bottom of Fig 1A.

French Guiana is a 84 000 km^2^ French oversea department located in South America, bordering on the northeast Atlantic coast between Suriname and Brazil at a latitude of 2–6° North (Fig 1A). The climate is equatorial with little variations in the monthly average temperature that stays around 25–26°C all year through, although the average high temperature increases from 28–29°C to 30–31°C in August-November. By contrast, rainfall that annually accounts for an average of 2 800 mm, varies strongly within year with rainy seasons of long (March-June) and short (December-January) durations, set apart by similarly long (July-November) and short (February) dry seasons [38,39]. The territory shows geological and topographic variations that lead to spatial heterogeneity in the nature of the soil. Those variations have allowed for a partition of French Guiana into geomorphologic landscapes (Fig 1A, [40]) that have been shown to be associated with the variations in plant [41] and vertebrate [42] communities. The Coastal Plain consists of a belt of marshy land and relatively low canopy with a dominance of Clusiaceae, Caesalpinioideae and Lecythidaceae developing on quaternary marine sediment. Behind this place, the land rises to higher mountains and plains. The Northern Chain is dominated by hills and a more diversified soil cover mixing clayic ferralsols with more sandy or loamy soils. The canopy is dominated by trees of the Lecythidaceae and Caesalpinioideae families. The central massif includes relatively flat relief of moderate elevation covered by well-drained ferralsols. Dominant tree families are Burseraceae, Mimosoideae and Caesalpinioideae, but one can also found palms. The Inini-Camopi chain is characterized by higher relief with many slopes and a high canopy and a large diversity of tree families that include many infrequent families such as Vochysiaceae, Malvaceae or Annonaceae. Finally, the Southern chain is characterized by very flat hills that remain partially inundated during the wet season. The canopy is typically low and discontinuous, with a high abundance of Burseraceae, Mimosoideae and Myristicaceae.

### Triatomine collections

Field collection of triatomines was carried out in 22 sites spread across 4 geomorphologic landscapes (Fig 1A, S1 Table). Because a large part of the French Guiana territory is hard to access, field sites were chosen both randomly and opportunistically, typically when boat or helicopter transportations were organized for large groups of scientists or local health/military authorities. The Southern chain was not sampled as this part of French Guiana mostly corresponds to uninhabited deep Amazonian forest. The 4 geomorphologic landscapes were labelled l = 1 (Coastal Plain), l = 2 (Northern Chain), l = 3 (Central Massif) and l = 4 (Inini-Camopi chain) to indicate their position on the North-South gradient that spread from the Atlantic coast to the Amazonian frontier with Brazil. Insects were captured using light traps located in the sylvatic environment during c_1_ = 20, c_2_ = 58, c_3_ = 93, and c_4_ = 85 nights spent all along the year in the 4 landscapes. Light traps consisted of a vertical white sheet lit by a 250 watts mercury vapour lamp, combined with a 400 watts lamp fixed a few meters above ground on a tower platform to increase attractiveness. According to the PLoS open access policy, these data are available on request. Specimens landing on the sheet were collected, placed in individual plastic vials and brought to the laboratory for species determination, using the systematic revision of [43], and molecular diagnosis of infection by *T*. *cruzi*.

### Molecular diagnosis of *Trypanosoma cruzi* infection

The infection of triatomines by *T*. *cruzi* was tested on 651 insects. We used Polymerase Chain Reaction (PCR) amplification of *T*. *cruzi* kinetoplast DNA (kDNA) from rectal content. DNA was extracted using the DNeasy blood and Tissue kit (Qiagen, Hilden, Germany), and amplified with the 330 bp variable region of kDNA using the following three primers; 121a (5’ AAATAATGTACGGGGGAGATGCATGA 3‘), 121b (5’ AAATAATGTACGGGTGAGATGCATGA 3’), and 122 (5’ GGTTCGATTGGGGTTGGTGTAATATA 3‘). The reaction mixtures contained 10–30 ng of extracted DNA; 250 μM of each desoxyribonucleotide triphosphate (dNTP) (Sigma), 2.5mM of MgCl2, 0.2 μM of each primer (Applied Biosystem) and 5 U/μL of HotStarTaq DNA Polymerase (Qiagen). The following PCR cycling were used: 94°C for 15 min; 35 cycles of 94°C for 1 min, 64°C for 30 s, and 72°C for 20 s; and 72°C for 5 min. The PCR products were revealed after electrophoresis on a 1.5% agarose gel stained with ethidium bromide (0.5 μg/mL) using an ultraviolet transilluminator. Negative controls included DNA sample from *Brontostoma colossus* (Reduviidae, non-hematophageous and uninfected control) and distilled water (non-DNA negative control).

### Data analysis

#### Geographic distribution of triatomines and their infection by *T*. *cruzi*

The spatial variations in triatomines’ overall abundance (A), species diversity (SD) and their infection by *T*. *cruzi* (ITc), were investigated through a model selection approach that allowed the evaluation of all hypothetical associations between A, SD, or ITc, and the geomorphologic landscapes.

#### Hypotheses and models

We identified 15 possible hypothetical associations and models that ranged from no heterogeneity between landscapes (model m = 1: 1 = 2 = 3 = 4) to a complete heterogeneity (model m = 15: 1≠2≠3≠4). Intermediate hypotheses accounted for either two (models m = 2 to 9) or three (models m = 6 to 14) different levels of A, SD, or ITc across the landscapes. Specifically, models 2–9 allowed for A, SD, or ITc to be the same in all but one landscape (e.g. 1 = 2 = 3≠4) or to be similar in two pairs of landscapes (e.g. 1 = 2≠3 = 4). In models m = 6 to 14, two landscapes were supposed to be similar, while the remaining two had their own levels of A, SD, or ITc (e.g. 1 = 2≠3≠4). When considering A data, the models were defined with respect to mean numbers m_.,l_ of triatomine individuals collected per night in landscape l. The set of model parameters, denoted **θ**_m_, was then identified using the m_.,l_ and the above relationships. Typically, for m = 1, all mean numbers were identical (∀l, m_.,l_ = m_.,._) and the model had only one parameter: **θ**_1_ = {m_.,._}, while for m = 15, all mean numbers were different and the model had 4 parameters: **θ**_15_ = {m_.,1_,m_.,2_,m_.,3_,m_.,4_}. When dealing with SD and ITc data, the models were defined with respect to the probabilities p_s,l_ of occurrence of species s in landscape l and with the rates of infection r_s,1_ of species s in landscape l, respectively. The sets of parameters of all models used to analyse SD and ITc data were identified by applying the equal/unequal relationships in the exact same way as for the A data. A one by one description of the 15 hypotheses and models applied to A, SD and ITc data can be found in Table A in S2 Table.

#### Fitting the models to data

The first step in the model selection approach consists of identifying the best fit of each of the models to the data. Each of the 15 models were then fitted to the A, SD, or ITc data observed across landscapes according to a log likelihood value that was defined as

$${\text{LLH}}_{\text{m}}=\sum_{\text{l}}\text{logp}\left(X_{\text{l}}=O_{\text{l}}|\theta_{\text{m}}\right)$$ (1)

where **X**_l_, **O**_l_ and **θ**_m_ are vectors containing statistical variables representing expected values in landscape l, observed values in landscape l, and the set of model (m) parameters to be estimated through the fitting process. The content of each of these (mathematical) vectors were defined specifically when working with A, SD and ITc data.

Overall Abundances (A). While fitting models to A data, the vector **X**_l_ contained a unique statistical variable N_.,l_ corresponding to the expected number of individuals of all species in landscape l, **O**_l_ contained the observed number N_.,l_ of individuals of all species in landscape l, and **θ**_m_ was a set of means of abundance m_.,l_ defined according to the hypothesis being modelled. All mean numbers included in **θ**_m_ were estimated by maximizing the LLH_m_ value with the probabilities p(**X**_l_ = **O**_l_|**θ**_m_) defined according to a Poisson distribution:

$$\text{p}\left(X_{\text{l}}=O_{\text{l}}|\theta_{\text{m}}\right)={\text{e}}^{-\mu_{.,\text{l}}}\frac{\mu_{.,\text{l}}{}^{{\text{n}}_{.,\text{l}}}}{{\text{n}}_{.,\text{l}}!}$$ (2)

where μ_.,l_ = c_l_m_.,l_ stands for the expected number of triatomines collected in landscape l according to the specific number of nights of collection c_l_, which allowed to account for the differences in sampling intensity between landscapes.

#### Species diversity (SD)

While fitting models to SD data, the vector **X**_l_ was made of several statistical variables N_s,l_ corresponding to the expected number of individuals of each species s in landscape l, the vector **O**_l_ contained the observed number n_s,l_ of individuals of each species s in landscape l, and **θ**_m_ was the set of probabilities p_s,l_ of occurrence of each species s in landscape l that were defined according to the hypothesis and model considered. All probabilities in **θ**_m_ were estimated by maximizing the LLH_m_ (Eq 1) with the probabilities p(**X**_l_ = **O**_l_|**θ**_m_) defined according to a multinomial distribution:

$$\text{p}\left(X_{\text{l}}=O_{\text{l}}|\theta_{\text{m}}\right)=\frac{{\text{n}}_{\text{l}}!}{{\prod^{\text{​}}}_{\text{s}}{\text{n}}_{\text{s},\text{l}}!}{\prod^{\text{​}}}_{\text{s}}{\text{p}}_{\text{s},\text{l}}^{{\text{n}}_{\text{s},\text{l}}}$$ (3)

where n_l_ = Σ_s_ n_s,l_ stands for the total number of individuals observed in landscape l. We used a multinomial (rather than a negative binomial distribution) because the variance to mean ratio in triatomine abundance was low.

#### Infection by *T*. *cruzi* (ITc)

We fitted the models to the ITc data for each triatomine species. The vector **X**_l_ then contained a unique statistical variable I_s,l_ corresponding to the expected number of infected individuals of species s in landscape l, **O**_l_ contained the observed number i_s,l_ of infected individuals of species s in landscape l, and **θ**_m_ was the set of rates of infection r_s,1_ of species s in landscape l that were defined according to the hypothesis and model considered. The rates of infection in **θ**_m_ were estimated by maximizing the LLH_m_ value with the probabilities p(**X**_l_ = **O**_l_|**θ**_m_) defined according to a Binomial distribution:

$$\text{p}\left(X_{\text{l}}=O_{\text{l}}|\theta_{m}\right)=\left(\begin{array}{c} {\text{n}}_{\text{s},\text{l}}\\ {\text{i}}_{\text{s},\text{l}} \end{array}\right){\left({\text{r}}_{\text{s},\text{l}}\right)}^{{\text{i}}_{\text{s},\text{l}}}{\left(1-{\text{r}}_{\text{s},\text{l}}\right)}^{{\text{n}}_{\text{s},\text{l}}-{\text{i}}_{\text{s},\text{l}}}$$ (4)

where $\left(\begin{array}{c} {\text{n}}_{\text{s},\text{l}}\\ {\text{i}}_{\text{s},\text{l}} \end{array}\right)$ stands for the binomial coefficient.

#### Comparisons between models

The second step in the model selection approach consists of comparing the 15 models according to their ability to fit to the data (as measured by the LLH obtained for the best fits) and with respect to the number of their parameters. Such comparisons can be made using the standard Akaike Information Criterion (AIC) that is calculated for each model as:

AIC = -2LLH + 2P (5)

P was set to the number N_*p*_ of parameters of the model when analysing A, and to N_*p*_(1 − (N_*p*_ + 1)/N)^−1^ when analysing the SD and ITc. In the second case, where *N* = Σ_*l*_ *n*_*l*_ refers to the total number of adult insects in the dataset, the correction factor was introduced to minimize the risk of over-fitting associated with ratio *N*/N_*p*_ lower than 40, which provided a ‘corrected’ AIC referred to as AICc [44].

The rationale behind these comparisons is to allow evaluating when the increase in the complexity of the models (in our case from model 1 to 15) efficiently improves the description of the data. The model with the lowest AIC is selected as the most parsimonious and the corresponding hypothesis as the one receiving the best support from the data. Further calculations of the weight of Akaike for each of the model

$$\omega_{\text{m}}=\frac{{\text{e}}^{-\left({\text{AIC}}_{\text{m}}-\text{min}\left({\text{AIC}}_{\text{m}}\right)\right)/2}}{\sum_{\text{m}}{\text{e}}^{-\left({\text{AIC}}_{\text{m}}-\text{min}\left({\text{AIC}}_{\text{m}}\right)\right)/2}}$$ (6)

allow giving a more explicit measurement of this support as those weights give the probability for each model to provide the best representation of the data.

#### Seasonal variations in the abundance and infection of triatomines

The pattern of temporal variation in the abundance of triatomines was characterized by calculating the average number of insects trapped per night of collection for each month of the year during which insects were collected for at least 10 nights. The prevalence of infection by *T*. *cruzi* was estimated for each month of the year for which we performed molecular diagnosis of *T*. *cruzi* infection on at least 10 individuals. Durbin-Watson auto-correlation test [45,46] ran on residuals against the annual mean showed strong seasonal variations in vectors’ abundance (DW = 1.56 < threshold value = 1.76, positive auto-correlation) but fail to detect any tendency in triatomines’ infection (threshold-inf = 1.68 < DW = 1.96 < threshold-sup = 2.32, no auto-correlation). We then focused our analysis on abundance data, and characterized the observed seasonal variations using a similar model selection approach as described above for spatial patterns.

#### Hypotheses and models

We considered 3 different models to describe a range of possible temporal tendencies. The simplest model accounted for a unique peak of high abundance that was described by a normal distribution with a mean m corresponding to the timing of the peak, and a standard deviation sd measuring the ‘standard duration’ of the season (model 1). The other two models included two seasons of high abundance as local populations reported increases in triatomine abundance both in February (during the so-called ‘short dry season’) and in September-October. Each peak of abundance was then modelled by a normal distribution and characterized by its mean timing (m_early_, m_late_) and ‘standard duration’ (sd_early_, sd_late_). We set up models with either two peaks of equal (model 2) or unequal (model 3) weights, as there was *a priori* no information about the relative amount of insects present during the early and late season. In model 3, the asymmetry between the two high seasons was described by complementary probabilities for triatomines to be collected in the early (p) or late (1-p) season. Accordingly, models 1 to 3 were defined with respect to the following set of parameters;

**θ**_1_ = {m,sd}, **θ**_2_ = {m_early_, sd_early_, m_late_, sd_late_} and **θ**_3_ = {m_early_, sd_early_, m_late_, sd_late_, p}, respectively.

#### Fitting the models to data

The 3 models were fitted to the abundance data observed through the different months of the year according to a log likelihood value that was defined as

$${\text{LLH}}_{\text{m}}=\sum_{\text{t}}\text{logp}\left(X_{\text{t}}=O_{\text{t}}|\theta_{\text{m}}\right)$$ (7)

where vector **X**_t_, **O**_t_ and **θ**_m_ contained a statistical variable corresponding to the number of individuals expected at time t, the number of observed individuals at that time, and the parameters defined as described above. As we performed our analyses on each triatomine species represented by more than 50 individuals, and on the pool made of the four main species of the vector community, the statistical variable included in **X**_t_ was either the expected number of individuals of species s at time t, N_s,t_, or the expected number of individuals of all species s at time t, N_.,t_. The observed numbers where denoted n_s,t_ or n_.,t_, and the set of parameters specified above described either species or community patterns. All these parameters were estimated by maximizing the LLH_m_ value with the probabilities p(**X**_t_ = **O**_t_|**θ**_m_) defined according to a Poisson distribution:

$$\text{p}\left(X_{\text{t}}=O_{\text{t}}|\theta_{\text{m}}\right)={\text{e}}^{-\mu_{\text{t}}}\frac{\mu_{\text{t}}{}^{{\text{n}}_{\text{t}}}}{{\text{n}}_{\text{t}}!}$$ (8)

where μ_t_ stands for the expected number of individuals collected at time t according to the specific number of nights of collection at time c_t_. Quantities appearing in Eq 8 were then set to μ_t_ = m_s,t_ c_t_ and n_t_ = n_s,t_ to analyse species s temporal variations, and to μ_t_ = m_.,t_ c_t_and n_t_ = n_.,t_ to deal with the seasonal variations of the abundance of the whole vector community.

#### Comparisons between models

Comparisons were made using the Akaike Information Criterion (Eq 5) where P was set to N_*p*_(1 − (N_*p*_ + 1)/N)^−1^ where *N* = Σ_*t*_ *n*_*t*_ refers to the total number of insects [44]. The weights of Akaike were calculated according to Eq 6 for each of the model.

#### Mathematical modelling of *T*. *cruzi* vectorial transmission

In order to quantify the impact of the spatial and temporal variation in the species abundance, diversity, and infection of triatomines that were identified on the risk of *T*. *cruzi* transmission to humans, we then use a ‘Force Of Infection’ (FOI) model (see [47] for a review). Although such measures lack a detailed description of socioeconomic factors, human behaviours and/or physiological status that can affect disease transmission, they are useful in ordering the risks to which populations are exposed in different places or periods of time. As such, they have been used for most vector-borne diseases including Chagas disease [47]. The FOI (λ) is defined as the probability for a susceptible host individual to acquire infection within a given time period. It is usually calculated from the (per susceptible host) number of potentially infectious contacts (PIC) with infected vectors of a given species, thereafter referred to as C, and the probability T of parasites transmission per PIC. We expanded this relationship to add up the number of PIC due to each vector species, and to account for their spatial and temporal variations. The FOI in landscape l and at time t is then given by:

$$\lambda_{\text{l},\text{t}}=\;1-{\left(1-T\right)}^{\sum_{s}{\text{C}}_{\text{s},\text{l},\text{t}}}$$ (9)

where the number of PIC due to species s present in landscape l and at time t is:

$${\text{C}}_{\text{s},\text{l},\text{t}}=\frac{{\text{n}}_{\text{s},\text{l},\text{t}}\;.{\text{ r}}_{\text{s},\text{l},\text{t}}\;.\text{ b }}{{\text{N}}_{\text{h}}}$$ (10)

with b and N_h_ denoting the vector biting rate and number of host individuals available for blood-meals, respectively.

These two quantities are commonly used to assess the risk of transmission for vector-borne diseases. In the case of Chagas disease, the stercorarian transmission of *T*. *cruzi* is associated with T values typically lower than 10^−3^ [25], so that those risk measures are directly proportional as long as the number of PIC remains lower than 10^2^. As this condition typically hold for intrusive vectors such as those present in French Guyana, we focused on the FOI to assess how the observed variations in vector diversity, abundance, and infection rate combined to define the spatial and temporal heterogeneity of the risk of transmission to humans.

## Results

### Geographical distribution of triatomines and their infection by *T*. *cruzi*

**Fig 2:**
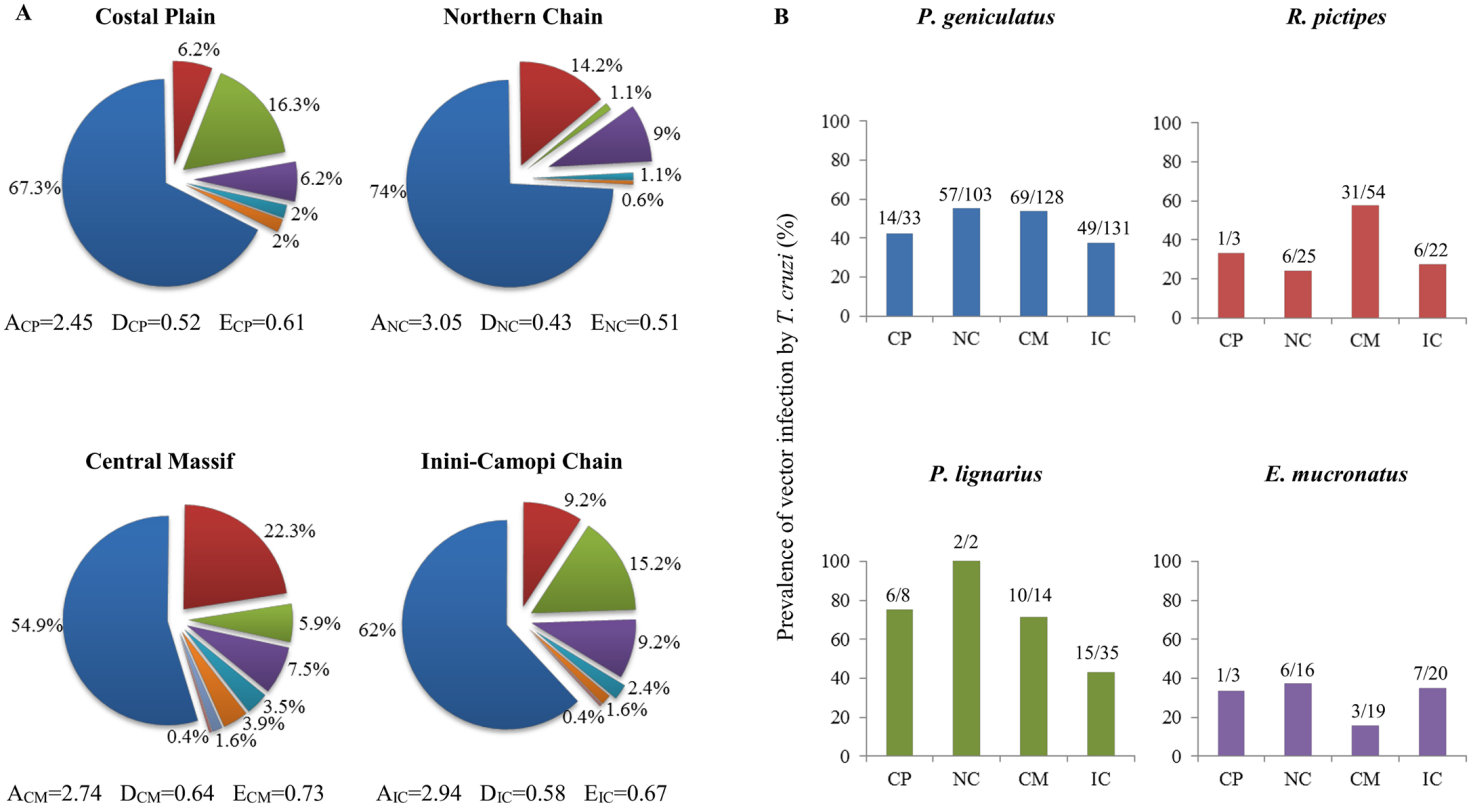
Geographical distribution of the biodiversity and infection of triatomine species in French Guiana. (A) Biodiversity of triatomines. Numbers appearing inside circles refer to the average number of individuals collected per night for each species. A, D and E and refer to the overall Abundance (per night of collection), Diversity and Equitability of the vector community. (B) Infection of the four main species of triatomines. Numbers appearing above each bar refer to the number of bugs infected with *T*. *cruzi* and the number of bugs tested for infection. The colour legend used to refer to each triatomine species is the same as in Fig 1.

The distribution of triatomines in French Guiana was investigated by collecting insects in 22 sites covering most of this territory (Fig 1A). A total of 731 adult specimens were captured during 256 nights of trapping. About 68.7% were males and 31.3% females. They belonged to 8 different species, with a dominant species (*Panstrongylus geniculatus*) representing 62.8% of the community, three secondary species (*Rhodnius pictipes*, *Panstrongylus lignarius*, *Eratyrus mucronatus*) whose abundances add up to 32%, and a set of four other species (*Panstrongylus rufotuberculatus*, *Rhodnius robustus*, *Rhodnius amazonicus*, and *Rhodnius paraensis*) that collectively account for the remaining 5.2% of the community (Fig 1B). The average number of triatomines collected per night ranged from 2.45 in the Coastal Plain (CP) to 3.05 in the Northern Chain (NC) with intermediate values of 2.74 and 2.94 in the Central Massif (CM) and the Inini-Camopi chain (IC), respectively (Fig 2A). To test for the significance of those geographical differences, we fitted the abundance data to our 15 models of spatial heterogeneity. The best model identified by the lowest AIC was model 1, in which the abundance of triatomines is evenly distributed across the four landscapes (Table 1, Table A in S2 Table). None of the other models that collectively accounted for all possible kinds of geographical heterogeneity was able to provide a better fit to the data despite their additional parameters. The Akaike weight of model 1 was 0.14, which was about twice as much as the weights of all other models, but models 4, 5 and 7 that reached 0.08–0.13 probabilities to be the best ones. Although these two more flexible models scored relatively well, the difference in abundance between landscapes they allowed for were actually estimated to be quite low when fitting them to the data. Accordingly, we conclude that the abundance of the triatomine community does not vary (or only marginally) across the four landscapes. The outcome of the selection model approach was quite different when considering the geographical distribution of triatomine species diversity (Table A in S2 Table). The best support went by far to model 11 that considered the same species distribution in CP and IC, but two landscape’s specific distributions in NC and CM. Even the most competitive alternatives received little support with weights of Akaike of about 10^−4^–10^−3^. On the contrary, the weight of Akaike associated to the best model was over 0.99, indicating a very strong support to the type of spatial heterogeneity in species distribution described in this model. Thus, although the abundance of the triatomine community does not seem to vary across landscapes of French Guiana, its species structure, and presumably its biodiversity, does so. We then used standard indexes of biodiversity (Exact Simpson D, e.g. [48,49]), Equitability E, e.g. [50], S2 Table) to characterize this spatial heterogeneity in vector communities, and found that the CP and IC chain show intermediate levels of diversity (D_CP_ = 0.52, D_IC_ = 0.58) and equitability (E_CP_ = 0.61, E_IC_ = 0.67) as compare to lower levels (D_NC_ = 0.43, E_NC_ = 0.51) in the NC, and higher levels in the CM (D_CM_ = 0.64, E_CM_ = 0.73). The low level of equitability in the NC corresponded to a strong dominance of *P*. *geniculatus* that represented 74% of the triatomine community, while the intermediate levels of equitability in CP and IC reflected a co-dominance of *P*. *geniculatus* (67.3% in CP and 62% in IC) and the three secondary species (28.7% in CP and 33.6% in IC) (Fig 2A). Finally, the CM showed the highest level of biodiversity as the abundance of *P*. *geniculatus* was further reduced (to 54.9%) while the frequency of the four species completing the assemblage increased to 9.4% (Fig 2A). Interestingly, the two landscapes with the lowest levels of biodiversity (as measured by either D or E) are those located in the northern part while the highest levels of biodiversity appeared in the two landscapes of the southern part, which suggest a coastal-inland gradient of triatomine biodiversity in French Guiana.

**Table 1:** Geographical distribution of the abundance and diversity of triatomine species.

Abundance					Diversity				
Model	LLH	AIC	Δi	ωi	Model	LLH	AICc	Δi	ωi
1: CP = NC = CM = IC	-15.1	32.1	0	0.1410	11: CP = IC vs NC vs CM	-41.9	133.4	0	0.9975
7: CP = CM vs NC = IC	-14.1	32.2	0.06	0.1370	8: CP = IC vs NC = CM	-57.3	147.3	13.8	9.9.10^−4^
5: NC = CM = IC vs CP	-14.4	32.8	0.69	0.0997	10: CP = CM vs NC vs IC	-48.9	147.5	14	9.1.10^−4^
4: CP = CM = IC vs NC	-14.6	33.1	1	0.0853	15: CP vs NC vs CM vs IC	-41.1	149.2	15.7	3.9.10^−4^

The four best models identified by the selection model approach are given with the associated LLH, AIC/AICc, differences between the lowest AIC and the AIC of each alternative (Δ_i_) and the weights of Akaike (ω_i_). The results obtained for the eleven other models that have been tested, but provided less supported predictions, are provided in Table A in S2 Table. AIC and AICc stand for Akaike Information Criteria and Akaike Information Criteria corrected to minimize the risk of over-fitting (see main text).

The spatial distribution of *T*. *cruzi* was investigated by molecular diagnosis of the infection performed on 651 of the 731 triatomines collected in the four sampled landscapes. We restricted our analysis to the four main vector species (Fig 2B) as we could not get enough individuals to confidently calculate the rates of infection in each landscape for the other species. For each of these four species the best model included some form of spatial heterogeneity; the best model was model 8 for *P*. *geniculatus*, model 3 for *R*. *pictipes* and *E*. *mucronatus*, and model 2 for *P*. *lignarius* (Table 2, Table B in S2 Table). For *P*. *geniculatus* the best model had twice as much support as any other model, while for *R*. *pictipes*, *P*. *lignarius* and *E*. *mucronatus* the best models had about 1.3, 1.7 and 1.3 (i.e. 30%, 70% and 30%) more support than their most competitive alternative that also included some form of spatial heterogeneity. Most importantly, the weights of Akaike of the above best models were 56, 75, 7.5 and 1.3 times higher than the weight of Akaike of model 1 that assumes no spatial variation in infection rates (Table B in S2 Table). There was thus a strong support from the data for the existence of spatial heterogeneity in infection with a geographical distribution that varied from one species to another; the prevalence of *T*. *cruzi* was found to be larger in NC and CM for *P*. *geniculatus*, in CM for *R*. *pictipes*, in CP, NC and CM for *P*. *lignarius* and in CP, NC and IC for *E*. *mucronatus* (see the definition of the best model for each species in Table 2, and Fig 2B).

**Table 2:** Geographical distribution of the infection of the four main triatomine species by *T*. *cruzi*.

Model	LLH	AICc	Δ_i_	ω_i_
***P*. *geniculatus***				
8: CP = IC vs NC = CM	-10	23.9	0	0.3375 (2, 56)
2: CP = NC = CM vs IC	-10.7	25.4	1.4	0.1656
1: CP = NC = CM = IC	-15	32	8	0.0060
***R*. *pictipes***				
3: CP = NC = IC vs CM	-6.5	17	0	0.2934 (1.3, 75)
7: CP = CM vs NC = IC	-6.8	17.6	0.58	0.2192
1: CP = NC = CM = IC	-11.8	25.7	8.6	0.0039
***P*. *lignarius***				
2: CP = NC = CM vs IC	-5.3	14.7	0	0.2378 (1.7, 7.5)
10: CP = CM vs NC vs IC	-4.7	15.7	1	0.1439
1: CP = NC = CM = IC	-8.3	18.7	4	0.0318
***E*. *mucronatus***				
3: CP = NC = IC vs CM	-5.5	15.3	0	0.1703 (1.3, 1.3)
7: CP = CM vs NC = IC	-5.8	15.7	0.46	0.1354
1: CP = NC = CM = IC	-6.9	15.8	0.53	0.1305

The two best models identified by the selection model approach, and model 1 that assumes no spatial variation, are given with the associated LLH, AICc, differences between the lowest AICc and the AICc (Δ_i_) and the weights of Akaike (ω_i_). In the last column, we reported within brackets the relative supports for the best model as compared to the second to the best and to model 1. The relative support was calculated as the ratio between the AICc of the best model and the AICc of the model it is compared to. The results obtained for the eleven other models that have been tested are provided in Table B in S2 Table. AICc denotes the Akaike Information Criteria corrected (see main text).

### Seasonal variations in the abundance and infection of triatomines

**Fig 3:**
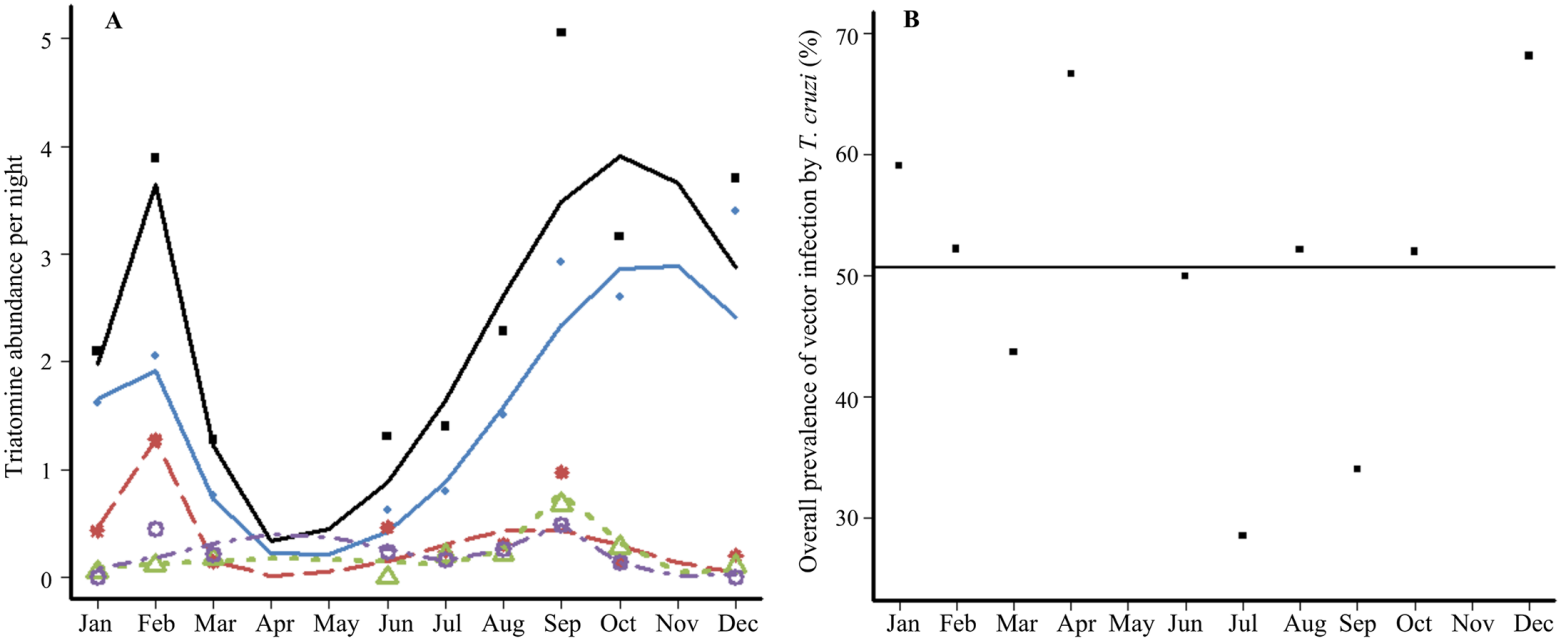
Seasonal variation in the abundance and infection of triatomine species in French Guiana. (A) Biannual variations in the abundance of triatomine species. The entire community appear in black (line and squares) and the other colours stand for the 4 main triatomine species: blue line and diamonds (*P*. *geniculatus)*, red line and stars *(R*. *pictipes)*, green line and triangles *(P*. *lignarius)*, *purple line and circles (E*. *mucronatus)*. (B) (Absence of) Seasonal variation in the overall rate of infection of triatomines.

The overall abundance of triatomines collected per night showed a strong pattern of seasonal variations (Fig 3A, black line). This pattern was best fitted by a model with two peaks of unequal importance that accounted for high abundances during an early period of short duration centred on February and during a longer late season in September-November. Those variations were negatively correlated with the monthly amount of precipitation (Spearman correlation coefficient = -0.72, N = 12, pvalue = 0.00551), but independent of the monthly average, minimal and maximal temperature (S3 Table). This bimodal pattern of seasonal variations was confirmed at the species level as, for each of the four main triatomine species, a model with two peaks always receive more support than the single peaked one (Table 3). Interestingly, while the early peaks of abundance of *P*. *geniculatus* and *R*. *pictipes* were in February, the early peaks of *P*. *lignarius* and *E*. *mucronatus* were shifted to April-May, coinciding with a time of the year when the two other species are the less abundant (Table 3 and Fig 3A). The late seasons of the four species were more synchronized, although they remained small differences in the timing of the peaks. Mostly, *P*. *geniculatus* showed a latter peak, centred on October-November, while the maximal abundances of the other three species were reached in August-September (Table 3 and Fig 3A). Overall, there was thus a marked biannual pattern in the variation of the abundance of each triatomine species with slightly different timing in the early/late seasons between species. On the contrary, the infection of vectors by *T*. *cruzi* showed no temporal trend (Fig 3B). The annual average prevalence of infection was 51% and, although there were some variations from one month to another that ranged between 29% in July and 69% in December, there was no sign of positive auto-correlation between those variations that would suggest a form of seasonality.

**Table 3:** Seasonal variations in the abundance of the triatomine community.

Model	LLH	AICc	Δ_i_	ω_i_	m_early_	sd_early_	m_late_	sd_late_
**Total vector community**								
A: 1 pic	-59.8	123.6	23.3	9.10^−6^	11.26	2.83		
B: 2 pics, p = 0.5	-53.1	114.3	14	9.10^−4^	1.71	2.05	9.57	1.53
**C: 2 pics, p = 0.13**	**-45.1**	**100.3**	**0**	**0.9990**	**2.71**	**0.36**	**10.63**	**2.36**
***P*. *geniculatus***								
A: 1 pic	-33.5	71	0.56	0.3827	11.36	2.48		
B: 2 pics, p = 0.5	-32.7	73.5	3.1	0.1100	0.99	2.09	9.98	1.79
**C: 2 pics, p = 0.07**	**-30.1**	**70.4**	**0**	**0.5073**	**2.85**	**0.19**	**11.05**	**2.26**
***R*. *pictipes***								
A: 1 pic	-49.7	103.4	25.7	10^−6^	12	4.2		
**B: 2 pics, p = 0.5**	**-34.7**	**77.7**	**0**	**0.5150**	**2.33**	**0.47**	**8.99**	**1.62**
C: 2 pics, p = 0.41	-33.6	77.8	0.12	0.4850	2.34	0.46	8.98	1.9
***P*. *lignarius***								
A: 1 pic	-22.4	49	6.2	0.0282	7.35	2.08		
**B: 2 pics, p = 0.5**	**-17**	**42.8**	**0**	**0.6287**	**4.87**	**2.81**	**9.6**	**0.58**
C: 2 pics, p = 0.64	-16.5	44	1.2	0.3430	6	3.68	9.63	0.49
***E*. *mucronatus***								
A: 1 pic	-25.8	61.2	6.5	0.0235	7	3.39		
B: 2 pics, p = 0.5	-20.2	55.8	1.1	0.3464	4.1	1.51	9.11	1.09
**C: 2 pics, p = 0.68**	**-18.2**	**54.7**	**0**	**0.6006**	**4.78**	**1.79**	**9.39**	**0.64**

The fit of each of the three models are presented with their respective LLH, AICc, difference with the lowest AICc, weight of Akaike (ω_i_) and the estimates of their parameters that allow characterizing the seasonal patterns. AICc denotes the Akaike Information Criteria corrected (see main text).

### Spatial and temporal variations in the risk of vectorial transmission of *T*. *cruzi*

**Fig 4:**
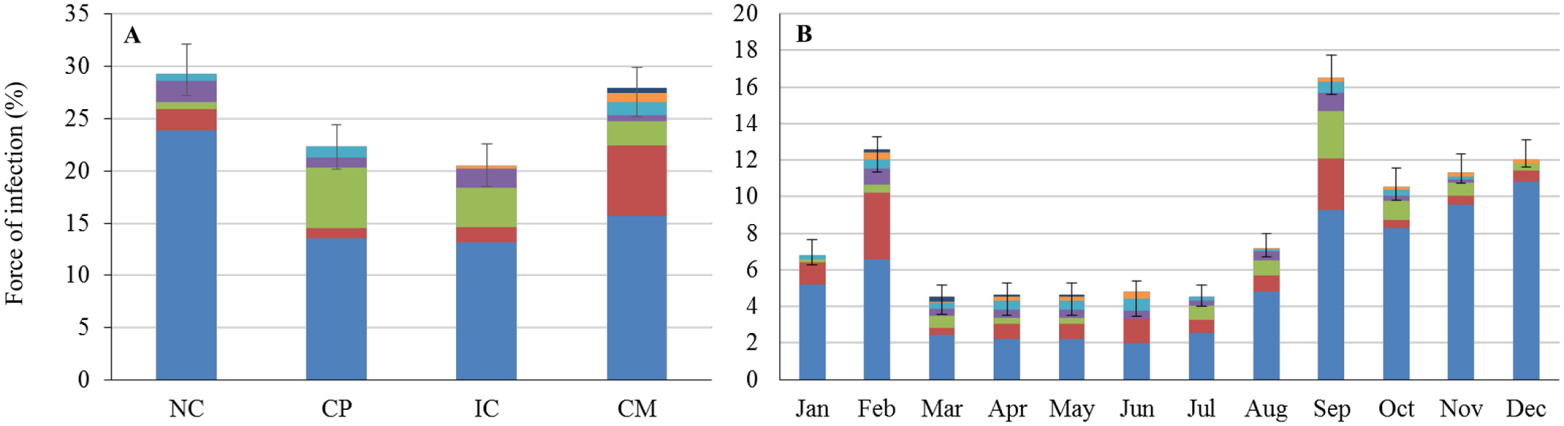
Spatial and temporal variations of the Force Of Infection (FOI) by *T*. *cruzi* in French Guiana. (A) Variations in the FOI by *T*. *cruzi* across the four landscapes ordered with respect to the observed level of triatomine biodiversity as measured by their D value (see Fig 2A). (B) Variations in the FOI by *T*. *cruzi* across the twelve months of the year. In Fig 4A and Fig 4B, the variations of the FOI are expressed as a percentage of the overall annual FOI. The colour legend used to refer to each triatomine species is the same as in Fig 1.

The heterogeneity in the geographical distribution of vector species and infection by *T*. *cruzi* led to spatial variations in the FOI (Fig 4A), with about 29.3%, 22.3%, 20.5% and 27.9% of the risk of transmission being located in NC, CP, IC and CM, respectively. Although those variations are not massive, they do exist and are statistically significant (χ^2^ = 42.6, df = 3, pvalue = 3.10^−9^). Interestingly, they reveal an unexpected relationship between the FOI and the biodiversity of the triatomine community, as shown in Fig 4A where landscapes are ordered according to their observed level of vector biodiversity. The less diverse triatomine community (in the Northern Chain) was associated with the highest level of risk of transmission mostly due to the dominant species, *P*. *geniculatus*, being concomitantly more abundant and infected with *T*. *cruzi* than in other landscapes. The increase in biodiversity substantially reduced the contribution of this key species in the three other landscapes. Meanwhile, the contribution of the secondary species and the species completing the assemblage increased. At intermediate levels of biodiversity (observed in the Coastal Plain and the Inini-Camopi chain) those latter contributions did not compensate for the reduced risk of transmission associated with the key species, so that the overall FOI decreased. However, in the most diverse community (in the Central Massif), their contributions further increased and nearly matched the contribution of the key species, *P*. *geniculatus*, so that the overall risk of vector transmission was larger than at intermediate levels of biodiversity. There also were temporal variations in the risk of infection. The biannual variations in species abundance were indeed well reflected in the monthly variations of the risk of *T*. *cruzi* transmission, which peaked both in February and September (Fig 4B). Overall, the 6-months period corresponding to these two peaks (i.e. September-February) accounted for about 70% of the risk of transmission with the remaining 6 months (March-August) represented only 30% of this risk.

## Discussion

The upper Amazonia and the Guyana shield form one of the three major tropical wilderness areas on earth, with a low human density (~3 people/km^2^) and one of the highest rates of population growth in the world (3.8%) [51]. The picture is very similar in French Guiana with very high levels of biodiversity [52], a low population density (~2.9 people/km^2^) and a strong annual growth rate (3.5%) due to both high fertility and immigration [53]. Such ecological and demographic attributes are likely to explain why these geographical areas have also been spotted as one of the ten hot-spots of Neglected Tropical Diseases [54]. Field studies carried out in French Guiana have suggested that these attributes could indeed influence dengue outbreaks [55], the endemic transmission of malaria [56] or the emergence of Buruli Ulcer [57], and they are identified as important risk factors for the emergence of an endemic situation of Chagas disease in French Guiana [58] and Amazonian Brazil [29]. An obvious research priority in such a context is to identify the spatial and temporal patterns of variations in the abundance and infection rate of species making up the vector community. This is not only necessary to gain a good understanding of the eco-epidemiology of the disease, which is still lacking in French Guiana [59], but also a critical pre-requisite to design the successful surveillance and control program intended by the local health authority [58].

### The biodiversity of triatomines in French Guiana and in Amazonia

The community of triatomines that can potentially transmit Chagas disease in French Guiana is a rich species assemblage made of a dominant species, *P*. *geniculatus*, representing 62.8% of the community; three secondary species, *R*. *pictipes*, *P*. *lignarius and E*. *mucronatus*, whose abundances add up to 32%, and a set of other species that collectively account for the remaining 5.2% of the community. Although we identified geographical variations in the species structure of this community (see below), the presence of one primary, three secondary and a set of other species, is remarkably stable and its level of diversity (D = 0.57 calculated on the total community) is 30% higher than the average level of diversity in other Amazonian assemblages of triatomine documented in the literature (D in 0.107–0.781 with an average of 0.44, S4 Table). This confirms that French Guiana is a hot-spot of triatomine biodiversity, as predicted by ecological niche models [60], and suggests that such models can be very useful in rationalizing the assessment of triatomine biodiversity, which remain under studied in many areas. This first quantitative assessment of the Chagas disease vector community in this area genuinely completes our current knowledge of the Amazonian biodiversity of triatomine species. The important abundance of *P*. *geniculatus* is consistent with entomological analysis of previous field and museum collections of triatomines in French Guiana [36,61,62], in close Surinam [63,64] and in other Amazonian places in Brazil [65–67]. Similarly, *R*. *pictipes*, *P*. *lignarius and E*. *mucronatus* have been described as parts of the community of triatomines in French Guiana [36,62], Surinam [64] and in Brazil [67–71]. The overall number of triatomine species in the Amazonia is probably 15–20 [72] with a local species richness typically larger than 10 in various Ecoregions [37]. As expected given such level of biodiversity, several species that have been identified as parts of the triatomine community in other Amazonian places did not appear in our collections, such as *Alberoprosenia malheiroi* [72–75], *Belminus laportei* [72,74–76], *Cavernicola lenti* [72,74,75,77], *R*. *brethesi* [72,75,78] et *T*. *maculata* [64]. Those species might or might not be present in French Guiana and we can only speculate that, if they are, one expects them to appear at low frequency. Finally, we note that five other species (*Panstrongylus megistus*, *Microtriatoma trinidadensis*, *Cavernicola pilosa*, *Panstrongulus mitarakaensis*, *Triatoma rubrofasciata*) have been observed in French Guiana in the past [36]. Their absence from our records suggests that they belong to the set of complementary species that add up to the key and secondary species to constitute the complete biodiversity of the Triatominae subfamily in this area.

### Variation in triatomine diversity and its relationship with *T*. *cruzi* infection

The species structure of the French Guiana triatomine community varies both in space and time. Although the abundance of the triatomine community itself, i.e. the total number of individuals captured per night, appeared to be very similar across the entire territory, species diversity did vary across the geomorphologic landscapes. The lower levels of vector diversity in the geomorphological landscapes located in the Northern part (Coastal Plain and Northern Chain) as compared to the biodiversity observed in the central and south parts (Central Massif and Inini-Camopi chain) suggest a coastal-inland gradient of triatomine biodiversity. Importantly, these spatial variations reveal a non-monotonic relationship between triatomine biodiversity and the risk of transmission to humans with intermediate diversity levels providing lower risks of transmission than both less and more diverse vector communities. Accordingly, vector biodiversity could either dilute (at the lowest diversity levels) or amplify (at the highest levels) the risk of transmission. Most of the theoretical studies on the effect of biodiversity on vector-borne diseases have been focused on the effect of host diversity on transmission while considering a single vector species (e.g. [79–81]). In a rare attempt at modelling vector communities, [82] found that a decrease in species richness would consistently reduce pathogen transmission. Such a theoretical reduction emerges as a result of a correlated decrease in the overall abundance of vectors. Here, we have shown that, in the absence of such correlation between biodiversity and overall vector abundance, the relationship between vector biodiversity and transmission can exhibit unexpected (non-monotonic) patterns. This suggests that evaluating the role of biodiversity on the transmission of vector borne-diseases, and the corresponding ecosystem services, requires to consider not only host biodiversity (as mainly done, see [10] for a review), but also potential changes in vector assemblages. When trying to delineate under what conditions disease risk is likely to decrease with host diversity (the so-called ‘dilution effect’), a key criteria is that host species more likely to be present or abundant in diverse community should reduce vector abundance [83]. Our results show that such criteria may not be appropriate for vector-borne diseases that are transmitted by a high diversity of vector species, such as malaria, African sleeping sickness or leishmaniasis [16]. In such cases, transmission would be diluted when the abundance of the most competent vector species decrease(s) with biodiversity, which, as observed in this study, does not need to be associated with a decrease in the abundance of the vector community.

Another important outcome of our study is that, although the abundance of the community remained similar across the four geomorphological landscapes, it did vary within year. We indeed provide here the first report of a strong biannual pattern of variation in the abundance of triatomine species with short early peaks in February-April and late broad peaks in August-November. These results provide a quantitative support to the oral reports made by the communities that during the ‘short dry season’ many triatomines were caught flying into houses. Such variations could be true seasonal changes in triatomine abundance or they could be due to variations in the rate of dispersal, either of which being potentially explained by the strong biannual pattern of rainfall observed in the area. Because the transmission of vector-borne pathogens is linearly connected to vector abundance (e.g. [25,34]), seasonal variations in the latter were expected to lead to temporal changes in vector infection rates. We could not detect any changes in the rate of triatomine infection by *T*. *cruzi*. This may be because the infection rates were measured in adult bugs that had already been accumulating infection over their life-time. Presumably, the assessment of *T*. *cruzi* prevalence in nymphal instars could reveal seasonal changes in the infection of the vector community. Although such developmental stages are very difficult to follow in the field, this would be worth evaluating to better understand the within year variation of *T*. *cruzi* transmission suggested by [67] in a similar entomological context.

### The risk of vector transmission of Chagas Disease in French Guiana

All bugs collected belonged to vector species described as primarily sylvatic [26], which reinforced the conclusion of previous studies which identified the triatomine species present in French Guiana [36]. Such vectors typically have a lower contact with humans than domesticated species, but they can still transmit *T*. *cruzi* through intrusions and transient infestations of the human habitat [see 26], which have been shown to be favoured by lights [84,85]. This risk of Chagas disease transmission is increasingly being documented, and has been shown to be associated with various triatomine species and with rates of *T*. *cruzi* infection in humans that reach 1–7% in Amazonian regions (e.g. [29,30]) and up to 16.8% in Mexico [32]. Populations of French Guiana are obviously exposed to the risk of Chagas disease transmission and the level of exposure (FOI) that have been estimated in this study could be responsible for a prevalence of infection in humans that stands around 2–3% (cases 6–7 in [25]). This is consistent with the current estimates of *T*. *cruzi* infection that range in 0–7% in different localities of French Guiana [30]. Interestingly, our calculations showed that one of the lowest risks of Chagas disease transmission is in the Coastal Plain, which is a rather encouraging prediction as 70–80% of the population live in this part of the territory [56]. The recurrent intrusion of adult bugs raises the issue of the potential domiciliation of these primarily intrusive vector species. Importantly, nymphal instars and colonies of *P*. *geniculatus*, have repeatedly been reported in peridomiciles and/or inside human dwellings [66,68,86–89] which, together with a reduced sexual size dimorphism in domestic environment [90], suggest a strong potential for the domiciliation of *P*. *geniculatus*. There is currently no evidence of *P*. *geniculatus* domiciliation in this area, but the high prevalence of the species in the vector community makes such potential eco-evolutionary process a substantial health concern that calls for a specific surveillance of *P*. *geniculatus* in peri-domestic and domestic habitats of French Guiana. Preliminary data have recently shown that *P*. *geniculatus* bugs were mostly infected by TcIII-TcIV, presumably because of their preferential relationship with armadillo species [91], while *Rhodnius* were predominantly associated with *Didelphis* and TcI [91,92].

The transient infestation of houses by intrusive vectors is a key challenge to achieving sustainable vector control (e.g. [93–95]). Conventional spraying of insecticide is ineffective in such entomological context, although strong seasonal variations of triatomine bug abundance could allow for a reduction of intervention frequency [96,97]. Such temporal optimization of control has been discussed for populations of *T*. *dimidiata* showing a unique three months peak of infestation that accounted for 60–75% of vector annual abundance [96,98], and to match seasonal variations of *T*. *infestans* abundance (e.g. [99,100]) or probability of establishment [101]. However, the high vector biodiversity and biannual patterns of triatomine bug abundance encountered in French Guiana (with 70% of the population of triatomines spread across a six-month period) makes it less likely that temporal optimisation would reduce intervention frequency. According to the efficacy of various strategies of insecticide spraying on similarly intrusive vectors [96], it is most likely that interventions would be required on an annual basis to substantially reduce disease risk in such context. This suggests that the need to consider alternative strategies based on physical or chemical barriers to control intrusion into human dwellings is even stronger in the Amazonian context than in other places where the risk of transmission is associated to only one or two intrusive vector species, such as *T*. *dimidiata* in the Yucatan peninsula, Mexico and Belize [102,103], *Rhodnius prolixus* in Venezuela [104], *T*. *mexicana* in central Mexico [105] or *T*. *brasiliensis* and *T*. *pseudomaculata* in Brazil [106]. Such barriers typically are provided by mosquito nets or impregnated curtains that have been shown to reduce triatomines house infestation by 80–95% for at least two consecutive years in pilot studies [85,107,108]. Such control interventions could be part of an integrated management as insecticide treated nets also represent sustainable options to protect the local populations against malaria, that is highly endemic in French Guiana [109], and outbreaks of dengue, that have been recurrently affecting the area for now 20 years [110,111]. This integrated management would be worthwhile in the context shaped by the current emergence of multiple resistances to insecticides in French Guiana [112].

### Conclusion

Our study has provided the first quantitative description of the spatio-temporal patterns of triatomine biodiversity and their infection by *T*. *cruzi* in French Guiana. Such knowledge is a critical step in developing eco-epidemiological studies of the transmission of Chagas disease by rich communities of intrusive triatomine species typically encountered in Amazonian areas. Along with a basic knowledge required for public health policy makers to better apprehend the transmission of *T*. *cruzi* in French Guiana, one of the 21 areas endemic for Chagas disease [22,23], we have provided the first report of a non-linear relationship between (vector) biodiversity and the risk of *T*. *cruzi* transmission. This is also the first report of biannual variation in bug abundance, exposing the population to a "double jeopardy" of annual infection. Smaller scale field studies of both the triatomine and the host community should now be combined with mathematical modelling of the vector spatial dynamics and *T*. *cruzi* transmission to improve our understanding of the effect of biodiversity on Chagas disease risk. Only such quantitative approach will allow identifying the service that biodiversity provides (or not) to dilute the transmission of Chagas disease. A full evaluation of vector species diversity will also allow optimizing the strategies towards transmission interruption despite the dual challenge set by Amazonian vector communities that are made of both highly diverse and mostly intrusive species.

## Supporting Information

S1 Table List of field sites and their spatial coordinates. (PDF)

S2 Table Complete outcomes of the model selection analyses and indices of biodiversity. (PDF)

S3 Table Correlation between triatomine abundance and basic environmental variables. (PDF)

S4 Table Review of the Amazonian biodiversity of Triatominae. (PDF)
