# Supplementary material for: Amazonian Triatomine Biodiversity and the Transmission of Chagas Disease in French Guiana: In Medio Stat Sanitas
Source: PLoS Negl Trop Dis. 2016 Feb 11;10(2):e0004427. doi: 10.1371/journal.pntd.0004427 (PMC4750908; doi:10.1371/journal.pntd.0004427)
Supplement: S1 Table — (PDF) [file pntd.0004427.s001.pdf]

Index	Field site	DMS (Degree, Minute, Second)	
		Latitude	Longitude
**Coastal plain**			
1	Saint Laurent du Maroni	N 5° 29' 44.002"	O 54° 1' 50.998"
2	Awala-Yalimapo	N 5° 41' 27.764"	O 53° 55' 56.657"
3	Iracoubo	N 5° 29' 46.2"	O 53° 12' 14.039"
4	Macouria	N 4° 55' 16.751"	O 52° 29' 21.087"
5	Cayenne	N 4° 55' 21.72"	O 52° 19' 36.84"
6	Matoury	N 4° 50' 49.92"	O 52° 19' 51.959"
7	Roura	N 4° 37' 42.384"	O 52° 16' 17.76"
8	Ouanary	N 4° 12' 32.76"	O 51° 40' 15.24"
**Northern chain**			
9	Kaw	N 4° 30' 52.667"	O 52° 3' 59.667"
10	Régina	N 4° 18' 34.842"	O 52° 8' 3.044"
11	Montsinéry-Tonnegrande	N 4° 43' 7.933"	O 52° 29' 21.087"
12	Apatou	N 5° 9' 14.041"	O 54° 20' 23.58"
**Central massif**			
13	Trinité	N 4° 35' 20"	O 53° 18' 1"
14	Bélizon	N 4° 16' 35.458"	O 52° 38' 35.382"
15	Saint Georges	N 3° 53' 20.04"	O 51° 48' 4.319"
16	Armontabo	N 3° 43' 54.0"	O 52° 19' 11.1"
17	Nouragues	N 4° 02' 2.268"	O 52° 40' 1.728"
**Inini-Camopi chain**			
18	Papaichton	N 3° 48' 25.2"	O 54° 8' 58.199"
19	Antecum Pata	N 3° 15' 0.751"	O 54° 6' 47.373"
20	Haut Koursibo	N 3° 43' 54.0"	O 53° 19' 11.1"
21	Saül	N 3° 51' 37.691"	O 53° 18' 15.402"
22	Camopi	N 3° 9' 55.8"	O 52° 20' 27.959"

Table : Field sites and their spatial coordinates.
