# Supplementary material for: Amazonian Triatomine Biodiversity and the Transmission of Chagas Disease in French Guiana: In Medio Stat Sanitas
Source: PLoS Negl Trop Dis. 2016 Feb 11;10(2):e0004427. doi: 10.1371/journal.pntd.0004427 (PMC4750908; doi:10.1371/journal.pntd.0004427)
Supplement: S2 Table — (PDF) [file pntd.0004427.s002.pdf]

	Abundance				Diversity			
**Model**	**LLH**	**AIC**	**Δ_i**	**ω_i**	**LLH**	**AIC_c**	**Δ_i**	**ω_i**
1 : CP=NC=CM=IC	**-15.1**	**32.1**	**0**	**0.1410**	-79.9	175.9	42.5	$5.9.10^{-10}$
2 : CP=NC=CM vs IC	-14.9	33.8	1.67	0.0610	-64.3	161.4	28	$8.3.10^{-7}$
3 : CP=NC=IC vs CM	-14.7	33.4	1.34	0.0722	-61.0	154.7	21.3	$2.4.10^{-5}$
4 : CP=CM=IC vs NC	-14.6	33.1	1	0.0853	-62.1	157	23.6	$7.6.10^{-6}$
5 : NC=CM=IC vs CP	-14.4	32.8	0.69	0.0997	-76.0	184.8	51.4	7.10^{-12}
6 : CP=NC vs CM=IC	-15.0	34.0	1.93	0.0537	-68.3	169.4	36	$1.5.10^{-8}$
7 : CP=CM vs NC=IC	-14.1	32.2	0.06	0.1370	-66.8	166.3	32.9	$7.3.10^{-8}$
8 : CP=IC vs NC=CM	-15.1	34.1	2	0.0520	-57.3	147.3	13.8	$9.9.10^{-4}$
9 : CP=NC vs CM vs IC	-14.7	35.4	3.31	0.0269	-50.8	151.2	17.8	$1.4.10^{-4}$
10 : CP=CM vs NC vs IC	-14.0	34.0	1.92	0.0540	-48.9	147.5	14	$9.1.10^{-4}$
11 : CP=IC vs NC vs CM	-14.5	34.9	2.81	0.0346	**-41.9**	**133.4**	**0**	**0.9975**
12 : NC=CM vs CP vs IC	-14.3	34.7	2.57	0.0390	-56.5	162.6	29.2	$4.7.10^{-7}$
13 : NC=IC vs CP vs CM	-13.8	33.6	1.52	0.0658	-59.0	167.6	34.2	$3.8.10^{-8}$
14 : CM=IC vs CP vs NC	-14.1	34.1	2	0.0518	-58.6	167	33.5	$5.2.10^{-8}$
15 : CP vs NC vs CM vs IC	-13.7	35.5	3.38	0.0260	-41.1	149.2	15.7	$3.9.10^{-4}$

Table A : Model selection approach applied to spatial variations in triatomines' overall abundance and diversity.

S2 Table (Péneau et al.)

Model	LLH	AICc	Δi	ωi
P. geniculatus				
1 : CP=NC=CM=IC	-15	32	8	0.0060
2 : CP=NC=CM vs IC	-10.7	25.4	1.4	0.1656
3 : CP=NC=IC vs CM	-13.6	31.2	7.3	0.0089
4 : CP=CM=IC vs NC	-13.4	30.9	6.9	0.0105
5 : NC=CM=IC vs CP	-14.8	33.6	9.6	0.0027
6 : CP=NC vs CM=IC	-14.2	32.4	8.5	0.0048
7 : CP=CM vs NC=IC	-14.2	32.5	8.6	0.0046
**8 : CP=IC vs NC=CM**	**-10**	**23.9**	**0**	**0.3375**
9 : CP=NC vs CM vs IC	-10.6	27.3	3.4	0.0623
10 : CP=CM vs NC vs IC	-10.5	27	3.1	0.0719
11 : CP=IC vs NC vs CM	-9.9	25.9	2	0.1252
12 : NC=CM vs CP vs IC	-9.8	25.7	1.8	0.1405
13 : NC=IC vs CP vs CM	-13.5	33.2	9.2	0.0034
14 : CM=IC vs CP vs NC	-13.4	32.8	8.8	0.0041
15 : CP vs NC vs CM vs IC	-9.8	27.7	3.7	0.0519
R. pictipes				
1 : CP=NC=CM=IC	-11.8	25.7	8.6	0.0039
2 : CP=NC=CM vs IC	-10.5	25.1	8	0.0053
**3 : CP=NC=IC vs CM**	**-6.5**	**17**	**0**	**0.2934**
4 : CP=CM=IC vs NC	-9.5	23	6	0.0147
5 : NC=CM=IC vs CP	-11.8	27.7	10.6	0.0014
6 : CP=NC vs CM=IC	-9.4	22.9	5.8	0.0160
7 : CP=CM vs NC=IC	-6.8	17.6	0.58	0.2192
8 : CP=IC vs NC=CM	-10.4	24.9	7.9	0.0057
9 : CP=NC vs CM vs IC	-6.4	19.1	2.1	0.1034
10 : CP=CM vs NC vs IC	-6.7	19.7	2.6	0.0785
11 : CP=IC vs NC vs CM	-6.4	19.1	2	0.1071
12 : NC=CM vs CP vs IC	-10.4	27	9.9	0.0020
13 : NC=IC vs CP vs CM	-6.4	19.1	2.0	0.1061
14 : CM=IC vs CP vs NC	-9.3	24.9	7.8	0.0059
15 : CP vs NC vs CM vs IC	-6.4	21.2	4.1	0.0372

Table B : Model selection approach applied to spatial distribution of the infection of the four main vector species by *T. cruzi*

S2 Table (Péneau et al.)

Model	LLH	AICc	Δi	ωi
P. lignarius				
1 : CP=NC=CM=IC	-8.3	18.7	4	0.0318
**2 : CP=NC=CM vs IC**	**-5.3**	**14.7**	**0**	**0.2378**
3 : CP=NC=IC vs CM	-7.4	19	4.3	0.0275
4 : CP=CM=IC vs NC	-7.1	18.5	3.8	0.0358
5 : NC=CM=IC vs CP	-7.6	19.4	4.7	0.0224
6 : CP=NC vs CM=IC	-6.8	17.8	3.1	0.0500
7 : CP=CM vs NC=IC	-6.3	16.8	2	0.0859
8 : CP=IC vs NC=CM	-6.6	17.5	2.8	0.0590
9 : CP=NC vs CM vs IC	-5.1	16.7	2	0.0883
10 : CP=CM vs NC vs IC	-4.7	15.7	1	0.1439
11 : CP=IC vs NC vs CM	-6	18.5	3.8	0.0363
12 : NC=CM vs CP vs IC	-5.3	16.9	2.2	0.0786
13 : NC=IC vs CP vs CM	-6.3	18.9	4.2	0.0289
14 : CM=IC vs CP vs NC	-6.3	19	4.3	0.0274
15 : CP vs NC vs CM vs IC	-4.6	18	3.3	0.0465
E. mucronatus				
1 : CP=NC=CM=IC	-6.9	15.8	0.53	0.1305
2 : CP=NC=CM vs IC	-6.6	17.5	2.2	0.0566
**3 : CP=NC=IC vs CM**	**-5.5**	**15.3**	**0**	**0.1703**
4 : CP=CM=IC vs NC	-6.5	17.2	2.0	0.0633
5 : NC=CM=IC vs CP	-6.9	17.9	2.7	0.0453
6 : CP=NC vs CM=IC	-6.5	17.2	1.9	0.0653
7 : CP=CM vs NC=IC	-5.8	15.7	0.46	0.1354
8 : CP=IC vs NC=CM	-6.6	17.4	2.1	0.0588
9 : CP=NC vs CM vs IC	-5.5	17.5	2.2	0.0566
10 : CP=CM vs NC vs IC	-5.7	17.9	2.7	0.0452
11 : CP=IC vs NC vs CM	-5.5	17.5	2.2	0.0570
12 : NC=CM vs CP vs IC	-6.6	19.6	4.3	0.0194
13 : NC=IC vs CP vs CM	-5.5	17.5	2.2	0.0565
14 : CM=IC vs CP vs NC	-6.5	19.4	4.1	0.0218
15 : CP vs NC vs CM vs IC	-5.5	19.7	4.5	0.0181

Table B : Model selection approach applied to spatial distribution of the infection of the four main vector species by *T. cruzi*

S2 Table (Péneau et al.)

Exact Simpson index:

$$D_l = 1 - \frac{1}{n_{.,l}(n_{.,l} - 1)} \sum_s [\, n_{s,l}\ (n_{s,l} - 1)]$$

where $n_{.,l}$ is the observed number of individuals of all species in landscape l, and $n_{s,l}$ the observed numbers of individuals of each species s in landscape l.

Equitability index:

$$E_l = \frac{D_l}{D_{l\ max}}$$

where D_l is the exact Simpson index value in landscape l and $D_{l\ max}$ the highest exact Simpson index value for the given number of species and the sample size in landscape l.

S2 Table (Péneau et al.)
