# Supplementary material for: Amazonian Triatomine Biodiversity and the Transmission of Chagas Disease in French Guiana: In Medio Stat Sanitas
Source: PLoS Negl Trop Dis. 2016 Feb 11;10(2):e0004427. doi: 10.1371/journal.pntd.0004427 (PMC4750908; doi:10.1371/journal.pntd.0004427)
Supplement: S3 Table — (PDF) [file pntd.0004427.s003.pdf]

**Month**	**Triatomine abundance** Rank (#/night)	**Rainfall** Rank (mm)	**Average Temperature** Rank (°C)	**Minimal Temperature** Rank (°C)	**Maximal Temperature** Rank (°C)	**Max-Min Temperature** Rank (°C)
**January**	7 (2.14)	4 (330)	12 (26.2)	5.5 (23.22)	12 (29.42)	11 (6.20)
**February**	2 (4.22)	7 (250)	11 (26.25)	4 (23.30)	11(29.48)	12 (6.18)
**March**	9 (1.48)	5 (260)	10 (26.6)	3 (23.44)	10 (29.88)	9 (6.44)
**April**	11 (0.40)	2 (360)	5 (26.85)	1 (23.68)	8 (30.1)	10 (6.42)
**May**	12 (0.35)	1 (440)	6.5 (26.8)	2 (23.54)	9 (30.06)	8 (6.52)
**June**	8 (1.62)	3 (335)	8.5 (26.7)	7 (22.92)	7 (30.32)	6 (7.4)
**July**	10 (1.44)	8 (195)	6.5 (26.8)	11 (22.46)	5 (30.92)	5 (8.46)
**August**	6 (2.33)	9 (130)	3 (27.4)	10 (22.50)	4 (31.62)	3 (9.12)
**September**	1 (5.30)	12 (50)	2 (27.5)	12 (22.40)	2 (32.3)	2 (9.9)
**October**	5 (3.33)	11 (55)	1 (27.65)	9 (22.60)	1 (32.56)	1 (9.96)
**November**	4 (3.70)	10 (125)	4 (27.2)	8 (22.84)	3 (31.88)	4 (9.04)
**December**	3 (3.80)	6 (255)	8.5 (26.7)	5.5 (23.22)	6 (30.46)	**7** (7.24)
**Rho**		-0.720	0.151	-0.469	0.364	0.315
**p-value**		0.0055	0.6801	0.0619	0.8816	0.8228

Table : Correlation between triatomine abundance (per night) and basic environnemental variables. The strength of the correlation was tested using algorithm AS 89 [1] implemented in function cor.test from R [2].

1. Best D, Roberts D. Algorithm AS 89: the upper tail probabilities of Spearman's rho. Applied Statistics. 1975:377-9.

2. R Development Core Team. R: A language and environment for statistical computing. R Foundation for Statistical Computing, Vienna, Austria. ISBN 3-900051-07-0, URL: http://www.R-project.org/. 2010
