# Supplementary material for: Amazonian Triatomine Biodiversity and the Transmission of Chagas Disease in French Guiana: In Medio Stat Sanitas
Source: PLoS Negl Trop Dis. 2016 Feb 11;10(2):e0004427. doi: 10.1371/journal.pntd.0004427 (PMC4750908; doi:10.1371/journal.pntd.0004427)
Supplement: S4 Table — (PDF) [file pntd.0004427.s004.pdf]

Triatomine community					*T. cruzi* infection
**Species**	**Nb**	**Habitat (sampling methodology)**	**D**	**E**	**Infection (positive/tested)**
French Guiana. This study					
P. geniculatus	459	Wild (light traps)	0.5692	0.6496	189/395
R. pictipes	108				44/104
P. lignarius	63				33/59
E. mucronatus	61				17/58
P. rufotuberculatus	18				9/14
R. robustus	16				5/15
R. amazonicus	4				2/4
R. paraensis	2				0/2
French Guiana [1]					
P. geniculatus	100	Outside/ Mixed environment (light traps)	0.7811	0.8560	No available data
R. pictipes	50				
P. lignarius	20				
E. mucronatus	40				
P. rufotuberculatus	20				
R. robustus	20				
R. amazonicus	5				
R. paraensis	3				
C. pilosa	3				
M. trinidadensis	1				
P. mitarakaensis	1				
State of Pará, Belem, Brazil [2]					
P. geniculatus	11	Mixed environment, mainly forest and some houses (light traps)	0.6921	0.7675	Qualitative data
R. pictipes	272				
P. lignarius	89				
E. mucronatus	55				
R. robustus	23				
P. rufotuberculatus	1				
R. paraensis	4				
M. trinidadensis	50				
B. herreri	4				
T. rubrofasciata	27				

Table : Review of the Amazonian biodiversity of *Triatominae.* For each published study, we reported the identified triatomine species, the number of individuals collected for each of them, the sampling environment and methodology. We use this information to calculate standard indices of biodiversity (Exact Simpson D, Magurran 2004 p. 114-115, Equitability E, Heip et al 1998), and we reported the data on *T. cruzi* infection when this was assessed.

Amazon rainforest, Brazil [3]					
P. geniculatus	38	Primary rainforest (light traps)	0.6796	0.7823	No available data
R. pictipes	2				
P. lignarius	9				
E. mucronatus	17				
P. rufotuberculatus	3				
R. robustus	5				
R. amazonicus	1				
Surinam [4]					
P. geniculatus	87	3 Museum collections	0.5584	0.6500	No available data
R. pictipes	268				
P. lignarius	3				
E. mucronatus	13				
P. rufotuberculatus	16				
R. robustus	41				
T. maculata	1				
P. geniculatus	14	Houses (rural environment, opportunistic sampling attracted by light)	0.4556	0.5665	No available data
R. pictipes	137				
P. lignarius	1				
P. rufotuberculatus	9				
R. robustus	30				
French Guiana [5]					
P. geniculatus	46	Forest (black light traps)	0.5841	0.7204	35/48
R. pictipes	11				
P. lignarius	1				
E. mucronatus	9				
P. rufotuberculatus	8				
Provincia de Jaén, Cajamarca Region, Peru [6]					
P. geniculatus	83	Houses (active research)	0.1095	0.136	No available data
R.robustus	4				
P. herrei	4721				
P. chinai	197				
R. ecuadoriensis	4				
P. geniculatus	12	Outside, house environment (active research)	0.0866	0.1297	
P. herrei	534				
P. chinai	13				

State of Pará, Belem, Brazil [7]					
P. geniculatus<br>*P. lignarius*	22<br>2	Burrow	0.5920	0.7359	65/118
R. pictipes<br>*R. robustus*	50<br>103	Palm			
M.trinidadensis	5	Bromeliads			
Furo do Rio Pau Grande Amazon Basin [8]					
P. geniculatus<br>*R. pictipes*<br>*P. lignarius*	21<br>7<br>4	Houses<br>(flashlight)	0.5222	0.7588	1/5<br>1/4
P. geniculatus<br>*R. pictipes*<br>*P. lignarius*<br>*E. mucronatus*	418<br>12<br>2<br>1	Pigisties<br>(flashlight)	0.0674	0.0897	20/118<br>0/3
P. geniculatus<br>*R. pictipes*<br>*P. lignarius*	9<br>10<br>1	Palm<br>(trees dissection)	0.5737	0.8175	2/9<br>3/10<br>0/1
Brazil [9]					
R. pictipes<br>*P. lignarius*<br>*R. robustus*<br>*M. trinidadensis*	535<br>49<br>553<br>14	Palm<br>(trees dissection)	0.5516	0.7349	No available data
Paço do Lumiar County, state of Maranhão, Brazil [10]					
R. pictipes<br>*P. lignarius*<br>*R. neglectus*	89<br>11<br>33	Palm trees<br>(trees dissection)	0.4875	0.7257	68%<br>27%<br>39%
R. pictipes<br>*P. lignarius*<br>*R. neglectus*	36<br>/<br>16	Houses<br>(capture by<br>household members)	0.4344	0.8521	28%<br>/<br>31%
Manaus, state of Amazonas, Brazil [11]					
P. geniculatus<br>*R. pictipes*<br>*R. robustus*	5<br>85<br>25	Mixed environment<br>Mainly forest area<br>(bait trap)	0.4081	0.4720	0/5<br>5/85<br>2/25
Tapajós River Region, State of Pará, Brazil [12]					
R. pictipes<br>*P. lignarius*<br>*R. robustus*	3<br>1<br>739	Palm trees<br>(trees dissection)	0.0107	0.0161	125/740
Ouro Preto do Oeste, State of Rondônia, Brazil [13]					
R. robustus<br>*R. prolixus*	459<br>350	Palm trees<br>(trees dissection)	0.1319	0.2633	176/494

1. Berenger J-M, Pluot-Sigwalt D, Pages F, Blanchet D, Aznar C. The triatominae species of French Guiana (Heteroptera: Reduviidae). Memorias Do Instituto Oswaldo Cruz. 2009;104(8):1111-6.

2. Miles MA, Desouza AA, Povoa M. Chagas disease in the Amazon Basin 3. Ecotopes of 10 Triatomine bug species (Hemiptera, Reduviidae) from the Vicinity of Belem, Para state, Brazil. Journal of Medical Entomology. 1981;18(4):266-78.

3. Castro MCM, Barrett TV, Santos WS, Abad-Franch F, Rafael JA. Attraction of Chagas disease vectors (Triatominae) to artificial light sources in the canopy of primary Amazon rainforest. Memorias Do Instituto Oswaldo Cruz. 2010;105(8):1061-4.

4. Hiwat H. Triatominae species of Suriname (Heteroptera: Reduviidae) and their role as vectors of Chagas disease. Memorias Do Instituto Oswaldo Cruz. 2014;109(4):452-8.

5. Dedet JP, Chippaux JP, Goyot P, Pajot FX, Tibayrenc M, Geoffroy B, et al. Natural hosts of Chagas-Disease in French Guiana - High-frequency of *Trypanosoma cruzi* zymodeme 1 in wild marsupialia. Annales De Parasitologie Humaine Et Comparee. 1985;60(2):111-7.

6. Cáceres AG, Troyes L, Gonzáles-Pérez A, Llontop E, Bonilla C, Heredia N, et al. Enfermedad de Chagas en la región nororiental del Perú. I. Triatominos (Hemiptera, Reduviidae) presentes en Cajamarca y Amazonas. Revista Peruana de Medicina Experimental y Salud Pública. 2002;19(1):17-23.

7. Mascarenhas BM, Sampaio Nunes de Mello S. Triatomíneos de Amazônia: ocorrência de triatomíneos na área do reservatório da Hidrelétrica de Tucuruí (Pará) e observaçóes sobre o ciclo evolutivo de Rhodnius robustos Larrousse, 1927 (Hemiptera, Reduviidae, Triatominae). Acta amazonica. 1986;16:607-15.

8. Valente VC, Valente SAS, Noireau F, Carrasco HJ, Miles MA. Chagas disease in the Amazon basin: Association of *Panstrongylus geniculatus* (Hemiptera : Reduviidae) with domestic pigs. Journal of Medical Entomology. 1998;35(2):99-103.

9. Miles MA, Arias JR, de Souza AA. Chagas' disease in the Amazon basin: V. Periurban palms as habitats of *Rhodnius robustus* and *Rhodnius pictipes* - Triatomine vectors of Chagas' disease. Memorias do Instituto Oswaldo Cruz. 1983;78(4):391-8.

10. Teixeira ARL, Monteiro PS, Rebelo JM, Arganaraz ER, Vieira D, Lauria-Pires L, et al. Emerging Chagas disease: Trophic network and cycle of transmission of *Trypanosoma cruzi* from palm trees in the Amazon. Emerging Infectious Diseases. 2001;7(1):100-12.

11. Fe NF, Magalhaes LK, Fe FA, Arakian SK, Monteiro WM, Vale Barbosa MdG. Occurrences of triatomines in wild and domestic environments in the municipality of Manaus, State of Amazonas. Revista Da Sociedade Brasileira De Medicina Tropical. 2009;42(6):642-6.

12. Stehling Dias FB, Quartier M, Diotaiuti L, Mejia G, Harry M, Lima ACL, et al. Ecology of *Rhodnius robustus* Larrousse, 1927 (Hemiptera, Reduviidae, Triatominae) in Attalea palm trees of the Tapajos River Region (Para State, Brazilian Amazon). Parasites & Vectors. 2014;7.

13. de Oliveira Meneguetti DU, Trevisan O, Aranha Camargo LM, Rosa RM. Natural infection of triatomines (Hemiptera: Reduviidae) by trypanosomatids in two different environments in the municipality of Ouro Preto do Oeste, State of Rondonia, Brazil. Revista Da Sociedade Brasileira De Medicina Tropical. 2012;45(3):395-8.
